# Canine and Feline Testicular Preservation

**DOI:** 10.3390/ani12010124

**Published:** 2022-01-05

**Authors:** Lúcia Daniel Machado da Silva

**Affiliations:** Laboratory of Carnivores Reproduction, Faculty of Veterinary Medicine, State University of Ceará, Fortaleza 60714-903, Ceará, Brazil; lucia.daniel.machado@hotmail.com or lucia.daniel@uece.br

**Keywords:** dog, cat, testes, conservation, vitrification, freezing

## Abstract

**Simple Summary:**

Testicular cryopreservation enables the maintenance of reproductive potential, the creation of germplasm banks and the transport of genetic material between different regions. This biotechnology represents the only possibility of preserving the fertility of prepubertal animals that have already died or that need to undergo gonadotoxic treatments. Despite advances in the use of cryopreserved testicular fragments, protocols that can be used in the clinical routine of dogs and cats have not yet been established. Due to the great importance of the topic, the objective of this review is to provide an overview of the subject, approaching the main works on testicular cryopreservation in dogs and cats.

**Abstract:**

The increased interest in breeding dogs and cats and their use as models for other canids and felids demand research to improve reproductive techniques. Among them, testicular cryopreservation stands out. Testicular cryopreservation enables the maintenance of reproductive capacity and allows the establishment of germplasm banks for several species of commercial value or at risk of extinction. Furthermore, it enables the transport of genetic material among different regions. It is noteworthy that this biotechnology represents the only possibility of preserving the fertility of prepubertal animals that have died, so it has great importance in the propagation of the genetic material of animals. The spermatogonia present in the testes can be cultivated in vitro and the sperm obtained can be used in artificial reproduction programs. Although advances have been achieved with the use of testicular fragments to obtain viable and functional germ cells, the establishment of protocols that can be used in clinical routine have not been concluded yet. The testicular cryopreservation process can be carried out through techniques such as slow freezing, fast freezing and vitrification. However, the protocols used for the canine and feline species are still in the experimental phase. Given the importance of the topic, the aim of this review is to draw a profile of the subject approaching the main works on testicular cryopreservation in dogs and cats.

## 1. Introduction

Testicular cryopreservation enables the maintenance of reproductive capacity [1,2,3,4] and allows the implantation of germplasm banks for several species of commercial value or even those at risk of extinction [5]. Moreover, it provides the transport of genetic material among different regions [2]. It is noteworthy that this biotechnology represents the only possibility of preserving the fertility of prepubertal animals that have died, and it thus has great importance in the propagation of their genetic material [6].

Spermatogonia present in the testes can be cultivated in vitro, and the sperm obtained in this manner can be used in artificial reproduction programs. Although advances have been achieved with the use of testicular fragments to obtain viable and functional germ cells, the establishment of protocols that can be used in clinical routine has not been concluded yet [7].

The testicular cryopreservation process can be carried out using techniques such as slow freezing, rapid freezing and vitrification. However, the protocols used for dogs and cats are still in the experimental phase.

## 2. Canine Species

There are very few published works about testicular cryopreservation in dogs. The pioneer work found on this subject in dogs dates back only to 2016 [8]. The main goal of this study was to evaluate the capacity of two different cryopreservation approaches (slow freezing versus vitrification) for testicular preservation in adult dogs. Table 1 provides a compilation of works about testicular cryopreservation in dogs.

### 2.1. Cryopreservation Method

Testicular cryopreservation represents an important tool to support assisted reproduction techniques [14]; in view of this, the immediate use of this biological material is not always applicable. Testicular cryopreservation can be performed using the conventional freezing method or vitrification [8,15]. The conventional method is characterized by slow freezing, whereas vitrification is characterized by being an ultra-fast freezing process [16].

When comparing the protocols of slow freezing and solid-surface vitrification, the latter revealed samples with a larger area composed of tubular compartment, tubular lumen, seminiferous epithelium and tunica propria. Furthermore, the intertubular compartment showed Leydig cells with normal morphology and typical characteristics of steroidogenic cells. These results showed that solid surface vitrification in association with dimethyl sulfoxide and trehalose was effective for histology and sperm ultrastructure preservation in adult dogs; however, viability assessment was not performed [8].

Recently, an article on testicular cryopreservation was published comparing the classical slow freezing versus needle-immersed vitrification methods in grey wolf (*Canis lupus*) testicular fragments [13]. The wolf is an animal of the same family as the dog. In this work, it was observed that the slow freezing method was better than the needle-immersed vitrification method in the morphological preservation of germ cells in this specie of wolf.

Testicular cryopreservation can be done from cell suspension or by fragments [17]. Cell suspension is a laborious and harmful method for cell proliferation and differentiation, and thus work with the testicles of dogs and cats has been carried out with fragments.

### 2.2. Fragment Size

Although a fragment’s size can influence the action of cryoprotective agents [18], thus far there are no studies with the aim of evaluating whether the size of the canine testicular fragment has an influence on the success of cryopreservation. On the other hand, such studies have already been carried out for cats.

In previous works, fragment sizes of 3 to 5 mm^3^ [8] and 0.4 × 0.4 × 0.4 cm [9,10,11] were used in testicular cryopreservation protocols for dogs.

### 2.3. Cryoprotectants

Dimethyl sulfoxide (DMSO) and glycerol (GLY) were tested separately using conventional freezing for testicular cryopreservation in adult dogs. The associations between dimethyl sulfoxide/ethylene glycol (EG) and glycerol/ethylene glycol were investigated in the vitrification process as well. A dimethyl sulfoxide/ethylene glycol combination provided better results than a combination of glycerol/ethylene glycol [11].

In conventional freezing, cryoprotectants were used separately. On the other hand, vitrification was performed using combinations of different cryoprotectants. This association in vitrification showed better results in preserving the seminiferous tubules compared to conventional freezing with a single cryoprotectant [11].

**Figure 1:**
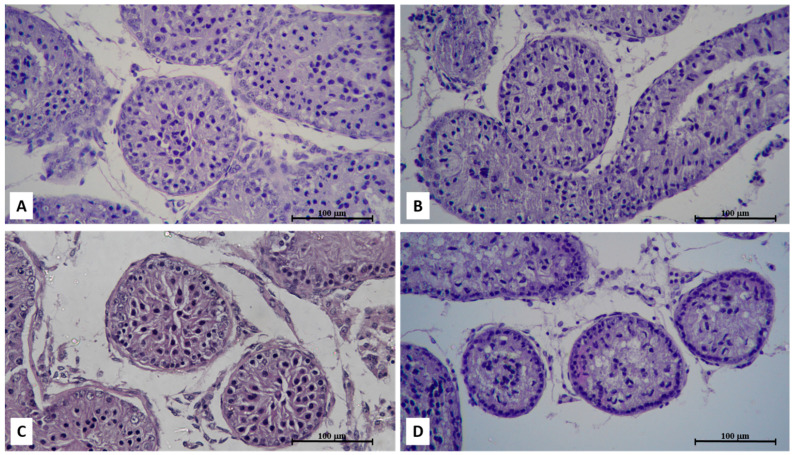
Photomicrograph of prepubertal dog testicular fragments. Note the poorly developed germinal epithelium, with good distinction between spermatogonia and Sertoli cells except in the last group. (**A**) Fresh seminiferous tubules with intact morphology; (**B**) Seminiferous tubules of testicular fragments vitrified with EG/GLY association, showing basement membrane retraction and nuclear visualization similar to the control; (**C**) Seminiferous tubules of testicular fragments vitrified with DMSO/EG association, showing greater separation of the basement membrane compared to the control; (**D**) Seminiferous tubules of testicular fragments vitrified with DMSO/GLY association, showing greater nuclear condensation and poor distinction between spermatogonia and Sertoli cells. H-E, 200×.

In our laboratory, the combination of cryoprotectants for testicular vitrification in prepubertal dogs is being evaluated to see which association best preserves testicular histological architecture. Thus far, preliminary results indicate that the EG/GLY and DMSO/EG associations are the ones that best preserve testicular integrity following the testicular vitrification process in prepubertal dogs (Figure 1). In all groups, the seminiferous tubules presented poorly developed germinal epithelium, with good distinction between spermatogonia and Sertoli cells, although the EG/GLY association was inferior to the other groups. In all vitrified groups, there was some degree of disorganization of the histological architecture, as demonstrated by the greater separation of the basement membrane compared to the control. All groups were similar to the control regarding basement membrane retraction and nuclear visualization. Regarding nuclear condensation, EG/GLY did not differ from the control, nor from the DMSO/EG, which in turn was inferior to the control and similar to DMSO/GLY [19].

### 2.4. Thawing/Warming

There was no work found in the literature about testicular cryopreservation in dogs with the purpose of testing different warming or thawing temperatures. In the works in which freezing was performed, three standard thawing protocols were carried out: (1) The cryopreserved testicular cells were thawed at 37 °C for 1 min [12]; (2) Straws were immersed into a water bath (37 °C) until the ice melted; the sealed end of the straws was cut, and the material drained into 2ml of the first thawing solution at 37 °C and incubated for 1 minute before materials were washed in the second solution at 37 °C for 5 min [8]; (3) Cryotubes were placed at room temperature for 30 s, then each fragment was placed in a warming medium (modified TCM 199 plus 20% FCS and 1M sucrose) for 10 min [9,10,11].

For post-vitrification warming, different protocols were adopted in the two studies found. The second was the same employed for thawing testicular fragments in the work of Santos (2018): (1) Cryovials containing vitrified testis fragments were kept at room temperature for approximately 30 s, then filled with 10 mL of solution 1 (Dulbecco’s modified Eagle’s medium nutriente mixture F-12—DMEM/F12 + 20% fetal bovine serum—FBS *v*/*v* + 100 mM trehalose) and the fragments were transferred into the same solution in a Petri dish at 37 °C with constant swirling for 2 min. Fragments were then transferred to a second dish containing solution 2 (DMEM + 20% FBS *v*/*v* + 50 mM trehalose) at room temperature for 1 min. All pieces then were washed twice in 5 mL DMEM + 20% FBS *v*/*v* each time with gentle swirling for 10 min at room temperature [8]; (2) Cryotubes were placed at room temperature for 30 s, then each fragment was placed in a warming medium (modified tissue culture medium 199—TCM 199 plus 20% FBS and 1 M sucrose) for 10 min [11].

### 2.5. Post-Xenotransplantation Spermatogenesis or In Vitro Culture

Protocols for sperm development through xenotransplantation have already been tested, and it could be an interesting alternative to test these protocols with cryopreserved testicular fragments. It was found that testicular fragments from immature animals xenotransplanted to immunocompromised mice were able to respond to endogenous gonadotropins, thus allowing complete differentiation of sperm capable of fertilization. Other studies have been carried out with this specific approach [20].

In this way, xenotransplantation of testicular fragments of two-month-old domestic dogs was carried out in the subcutaneous space of mice. After 13 months of culture, sperm was retrieved from 5 of the 29 fragments that were implanted [14].

Leaving the field of testicular fragments, testicular cells from Belgian Shepherd Malinois dogs four or five months in age were frozen, thawed after three months and cultivated in StemPro-34 medium. Then, colonies derived from germ cells and somatic testicular cells conjugated with extracellular matrix were transplanted into immunodeficient mice. The transplanted cells colonized the recipient testes, forming seminiferous tubules mainly composed of Sertoli cells and some germ cells. It was concluded that the StemPro-34 medium with dimethyl sulfoxide was optimal for the cryopreservation of canine testicular cells; the characteristics of the germ cells were maintained in the culture of colonies derived from germ cells cultivated after thawing, and the colonies derived from germ cells from transplanted dogs were able to colonize recipient mouse seminiferous tubules [12].

## 3. Feline Species

Table 2 shows a compilation of the works found in the literature regarding testicular cryopreservation in cats.

### 3.1. Cryopreservation Method

Testicular fragments from pubescent cats were used to compare slow freezing and rapid freezing, using dimethyl sulfoxide, ethylene glycol, glycerol and propanediol as cryoprotectants. The group subjected to slow freezing had significantly lower percentages regarding the degree of intact sperm plasma membrane when compared to the control group, which tended to be less than that of the two-step freezing technique (45.9% vs. 60.3% vs. 55.0%). On the other hand, the proportion of testicular spermatozoa with fragmented DNA was not different among the two freezing techniques and fresh testicular fragments. The fertilizing capacity of sperm was also demonstrated; after thawing, in vitro fertilization of mature oocytes was carried out and there was a progression to the blastocyst stage in 14% of cleaved embryos. This rate was similar to the one in control group [22].

### 3.2. Fragment Size

Fragment size considerably influences the action of the cryoprotective agent during freezing and/or vitrification [18]. Considering that testicular fragment size can be an important factor in cryopreservation, studies were carried out to test the influence of fragment size on feline testicular quality post-cryopreservation.

Fragments with 0.5 cm^3^ of volume were compared to 0.3 cm^3^ fragments. Larger fragments (0.5 cm^3^) had less harmful effects on germ cells than smaller ones (0.3 cm^3^) [23].

DNA damage was evaluated and apoptosis rates were estimated in testicular fragments obtained from cats after thawing. The values of these variables were compared regarding the type of cryoprotectant used (3% propanediol and 3% glycerol) and the size of the testicular fragment (0.3 cm^3^ and 0.5 cm^3^). The evaluation with acridine orange indicated that glycerol was more effective than propanediol in preserving DNA in cryopreserved 0.5 cm^3^ fragments. The results of the histomorphological evaluations indicated greater cellular integrity based on the evaluated criteria among the non-cryopreserved germ cells for both fragment sizes. The values of these variables decreased after cryopreservation, with no differences regarding the size of the stored fragment or cryoprotectants. In this work, immunohistochemical analysis using caspase-3 was conducted to investigate whether cellular apoptosis in the cytoplasm, nuclei or sperm occurred. After cryopreservation, both fragment sizes had similar percentages of samples with caspase-3 staining, with no particularly relevant findings for either cryoprotectant. This analysis showed differential staining between the cytoplasm and nuclei of seminiferous tubular cells along with the staining of spermatozoa heads at different intensities. The immunohistochemical results indicate that there was marked damage to all tissue fragments [26].

### 3.3. Cryoprotectants

The use of different cryoprotectants such as glycerol and propanediol has been proposed in the rapid freezing of cat testis. However, the histomorphological characteristics are better-preserved using glycerol [23].

Due to the need for high concentrations of cryoprotectants for vitrification, the association of cryoprotectants can be an alternative to reduce both the amounts of these concentrations as well as their harmful effects. In this way, the two-by-two association of the cryoprotectants dimethyl sulfoxide, ethylene glycol and glycerol were tested in the solid surface vitrification of testes of prepubertal cats. The combination of dimethyl sulfoxide/glycerol was the one that provided better conservation of the morphology of the seminiferous tubules (i.e., the smallest number of tubules seminiferous with separation and retraction of the basement membrane) and higher percentage of cell proliferation potential after warming [24].

When using the technique of vitrification in cryotubes with testicular fragments from prepubertal domestic cats, the effect of different combinations of cryoprotectants on cell viability after warming the fragments was evaluated. The dimethyl sulfoxide/glycerol combination was the only one to present a cell survival rate equal to that of the fresh group [25].

### 3.4. Thawing/Warming

Comprehending the importance of warming and reanimation conditions is essential to improve the survival of post-vitrification testicular cells. The structural and functional testicular properties of prepubertal domestic cats were studied after vitrification followed by two warming protocols (directly at 37 °C or with a pre-exposure of 5 s at 50 °C) and three reanimation moments (immediately, 24 h and 5 days post-warming). Preservation of the seminiferous tubule structure was better with warming at 50 °C for 5 s, and somatic and germ cell survival was greater compared to direct warming at 37 °C for one minute. Short-term in vitro culture (for resuscitation) proved that cell composition and functionality were better preserved when heated for a short period of time at 50 °C [7].

**Figure 2:**
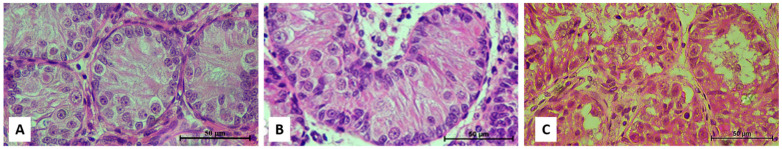
Photomicrograph of prepubertal cat testicular fragments. (**A**) Fresh seminiferous tubules with intact morphology; (**B**) Seminiferous tubules of testicular fragments warmed at 50 °C after vitrification using dimethyl sulfoxide/glycerol association, showing preserved morphology; (**C**) Seminiferous tubules of testicular fragments warmed at 60 °C after vitrification using dimethyl sulfoxide/glycerol association, showing degenerate morphology. Note the stroma rupture and basement membrane detachment. H-E, 400×.

In another work, three different warming temperatures (50, 55 and 60 °C for 5 s) were tested using the fresh testicular fragments as a control (Figure 2A). The aim of this work was to evaluate the possible influence of different warming temperatures on the structure, metabolic activity, composition, and cellular functions of the vitrified testicular fragments of prepubertal cats [27]. The authors concluded that vitrified testicular fragments from prepubertal cats have better preserved morphology, morphometry and viability when warmed at 50 °C (Figure 2B) compared to 60 °C (Figure 2C). This work sought to contribute to the knowledge regarding warming of testicular fragments after vitrification, reinforcing the importance of this step to the germplasm preservation process.

### 3.5. Cryopreserved Testis Xenograft

Xenotransplantation has been used as a form of ex situ cell culture in different species. This technique allows the obtaining of sperm cells from prepubertal animals. However, this method is still restricted to the experimental field.

In this way, slow freezing with dimethyl sulfoxide of testicular fragments from prepubertal and pubescent cats was carried out, followed by subsequent xenografts in immunosuppressed mice. After 10 weeks, it was observed that material from prepubertal cats showed an increase in the amount of stem cells and the presence of seminiferous tubules compared to that of pubescent cats, demonstrating that the testis of prepubertal animals had greater spermatogenic potential than that of pubescent animals [21].

## 4. New Approaches

Different cryopreservation techniques invariably lead to cellular cryoinjuries. To deal with these questions, researchers have been inspired to use anhydrobiosis to develop room temperature storage methods. Anhydrobiosis is the temporary suspension of vital activities that enable an organism to tolerate long dehydration. This is a natural process used by several small organisms to resist dry conditions. It is based on the properties of trehalose, which reaches a high concentration of solute without molecular mobility during dehydration, thus avoiding intra- and extracellular degradation.

Based on the principle of anhydrobiosis, one work has been presented using the cat as a model to develop future preservation at nonfreezing temperatures. The aim of this study was to characterize changes in histology, DNA integrity, and viability of adult testis versus prepubertal individuals during microwave-assisted drying. The results demonstrated for the first time that normal morphology, incidence of degeneration, DNA integrity and viability of testis remained at acceptable levels during microwave-assisted drying for 20 min. Overall, prepubertal testis appeared to be more resilient to microwave-assisted desiccation than adult testis [28].

Although the few studies on canine and feline testicular cryopreservation have been carried out using testicular fragments, in 2020, a German team published a paper on cryopreservation of a cat testicular cell suspension. The aim of this work was to establish a cryopreservation protocol for testicular cell suspension of domestic cats to be implanted in endangered feline species, applying two concentrations of dimethyl sulfoxide (7.5 and 15%) and performing a slow and a fast freezing protocol. The best protocol was obtained with slow freezing using 7.5% dimethyl sulfoxide, resulting in a mean cell survival rate of 45.4 ± 9.1% [29].

In 2021, a published work suggested epididymal vitrification as an alternative to the conservation of the male gamete as a means of preserving individual reproductive potential. The purpose of the study was to determine the effect of the vitrification of epididymal cauda by comparing the effects of glycerol and ethylene glycol on epididymal sperm quality post-vitrification. The authors observed that epididymal tail vitrification appears to be a suitable method for long-term storage of cat sperm, especially if the procedure is performed with ethylene glycol as the cryoprotectant [30].

## 5. Final Considerations

All cryopreservation techniques have risks of damaging cell structures, resulting in decreased cell viability. Despite the advances obtained, there is still no standardized optimal technique for cryopreservation, which remains an area of open study.

After reviewing some existing works on testicular cryopreservation in dogs and cats, it is observed that there is a divergence between the degrees of evolution in these two species. Undoubtedly, testicular cryopreservation in cats is more advanced than in dogs.

However, as interest in the area of cryobiology increases, especially testicular cryopreservation, the development and improvement of this area will certainly expand, contributing not only to the increased reproduction of dogs and cats, but to its application in wild canids and felids as well.

## Figures and Tables

**Table 1:** Papers addressing testicular cryopreservation in dogs.

Objectives	Technical Approach	Main Outcomes	Reference
Compare different cryoprotectants and compare conventional freezing with testicular vitrification.	Slow freezing and solid surface vitrification using DMSO, GLY, EG and trehalose.	Solid surface vitrification in association with DMSO and trehalose generated better results.	[8]
Evaluate the efficiency of DMSO and EG regarding the sperm membrane integrity after cryopreservation.	Slow freezing using DMSO or EG.	Both cryoprotectants were similarly efficient.	[9]
Compare two times of immersion in freezing medium and evaluate the effect on sperm membrane integrity.	Slow freezing using 15 or 30 min of immersion times.	Both the immersion times and the freezing technique used were efficient.	[10]
Compare different cryoprotectants and compare conventional freezing with testicular vitrification.	Slow freezing and solid surface vitrification using DMSO and EG isolated or in association.	Slow freezing using DMSO/EG trehalose generated better results.	[11]
Identify the optimal conditions for freezing canine testicular cells for GDCs culture, and to determine the spermatogonial stem cells capacity of these GDCs.	Slow freezing and in vivo or in vitro culture	StemPro-34 SFM as culture medium and DMSO as cryoprotectant were adequate for testis freezing, providing cells viable to develop during both in vivo and in vitro culture.	[12]
Histologically evaluate the testicles of prepubertal dogs vitrified with different associations of cryoprotectants	Solid surface vitrification using DMSO/GLY or DMSO/EG or GLY/EG	DMSO/EG and EG/GLY association were the ones that best preserved testicular integrity	[13]

DMSO: dimethyl sulfoxide; GLY: glycerol; EG: ethylene glycol; GDCs: germ-cell derived colonies.

**Table 2:** Papers addressing testicular cryopreservation in cats.

Objectives	Technical Approach	Main Outcomes	Reference
Focus on testis collection, cryopreservation and storage on ice-cold medium of testis fragment in an attempt to optimize conditions for posterior application to endangered felids.	Refrigeration at 4 °C or slow freezing using DMSO	Testicular cryopreservation with DMSO failed to produce grafts with germ cells. In contrast, testis from prepubertal animals may be preserved in ice cold medium for 2 to 5 days while pubertal testis showed signs of greater susceptibility to hypoxia/culture medium storage. Testis weight may be used to predict xenograft success, help decide the number of mice to use.	[21]
Determine the effects of CPAs and freezing protocols on testicular sperm plasma membrane and DNA integrity, and the fertilizing ability after ICSI	Two-step freezing vs. controlled slow freezing GLY, EG, PRO or DMSO	Testes were successfully cryopreserved. Types of CPAs and freezing techniques play a central role in determining the post-thaw quality of feline testicular spermatozoa. Frozen-thawed testicular spermatozoa retain fertilizing ability, although the development capability of embryos derived from ICSI with frozen-thawed testicular sperm is poor.	[22]
Evaluate testicular cryopreservation comparing two fragment sizes (0.3 and 0.5 cm^3^) and two cryoprotectants (GLY 3% and PRO 3%).	Conventional freezing	GLY was more efficient than PRO for cryopreservation and 0.5 cm^3^ fragments showed better results than 0.3 cm^3^ fragments.	[23]
Evaluate the effect of different associations of cryoprotectants on testicular integrity and the potential of spermatogonial proliferation after prepubertal testicular vitrification.	Vitrification using DMSO/GLY or DMSO/EG or GLY/EG	The association DMSO/GLY showed best testicular preservation and of the potential for cell proliferation after the vitrification.	[24]
Study structural and functional properties of testicular fragments from prepubertal cats after vitrification followed by two warming protocols (directly at 37 °C or with a 5-s pre-exposure to 50 °C) and three reanimation time points (immediately, 24 h and 5 days post-warming)	Vitrification	Preservation of seminiferous tubule structure was better using warming at 50 °C/5 s, and survival of somatic as well as germinal cells was higher compared to direct warming at 37 °C/1 min. Short term in vitro culture also proved that cellular composition and functionality were better preserved when warmed for a short time at 50 °C. Short warming at 50 °C led to better quality of seminiferous tubule structure and cell composition after vitrification and short-term culture.	[7]
Evaluate the effect of different associations of cryoprotectants on the testicular integrity, the potential for cell proliferation, and the viability of germ cells after in cryotubes	Vitrification using cryotubes	Vitrification in cryotubes can be successfully used for the testicular cryopreservation, and the DMSO/GLY association contributed the most to the maintenance of testicular histomorphological characteristics after vitrification.	[25]
Evaluate the damage to DNA and estimate the apoptosis rates in testicular fragments. Values for these variables were compared between type of cryoprotectant used (3% GLY and 3% PRO) and the size of the testicular fragment (0.3 and 0.5 cm^3^).	Conventional freezing	Both GLY and PRO at a concentration of 3% provided protection against damage caused by cryopreservation for both sizes of fragments. However, there were differences in the efficacy of the cryoprotectants regarding protection capacity depending on the type of the cell within the tissue.	[26]
Evaluate the influence of different warming temperatures (50, 55 and 60 °C) on the structure, metabolic activity, composition, and cellular functions of the vitrified testicular fragments of pre-pubertal cats.	Vitrification using DMSO/GLY	Vitrified testicular fragments from prepubertal cats have better preserved morphology, morphometry and viability when warmed at 50 °C.	[27]

DMSO: dimethyl sulfoxide; EG: ethylene glycol; GLY: glycerol; PRO: propanediol.

## Data Availability

This article is a literature review, therefore, all data described here were obtained from the works cited in the references.
